# Rosiglitazone Promotes Bone Marrow Adipogenesis to Impair Myelopoiesis under Stress

**DOI:** 10.1371/journal.pone.0149543

**Published:** 2016-02-19

**Authors:** Wenyi Lu, Weimin Wang, Shujuan Wang, Yonghuai Feng, Kaiyan Liu

**Affiliations:** 1 Department of Hematology, Peking University People’s Hospital, Beijing, China; 2 Institute of Hematology, Peking University, Beijing, China; 3 Beijing Key Laboratory of Hematopoietic Stem Cell Transplantation, Beijing, China; 4 Collaborative Innovation Center of Hematology, Peking University, Beijing, China; St. Vincent's Institute, AUSTRALIA

## Abstract

**Objective:**

The therapeutic use of thiazolidinediones (TZDs) causes unwanted hematological side effects, although the underlying mechanisms of these effects are poorly understood. This study tests the hypothesis that rosiglitazone impairs the maintenance and differentiation of hematopoietic stem/progenitor cells, which ultimately leads to hematological abnormalities.

**Methods:**

Mice were fed a rosiglitazone-supplemented diet or a normal diet for 6 weeks. To induce hematopoietic stress, all mice were injected once with 250 mg/kg 5-fluorouracil (5-Fu) intraperitoneally. Next, hematopoietic recovery, hematopoietic stem/progenitor cells (HSPCs) subsets, and myeloid differentiation after 5-Fu treatment were evaluated. The adipogenesis induced by rosiglitazone was assessed by histopathology and oil red O staining. The effect of adipocytes on HSPCs was studied with an *in vitro* co-culture system.

**Results:**

Rosiglitazone significantly enhanced bone marrow adipogenesis and delayed hematopoietic recovery after 5-Fu treatment. Moreover, rosiglitazone inhibited proliferation of a granulocyte/monocyte progenitor (GMP) cell population and granulocyte/macrophage colony-stimulating factor (GM-CSF) colonies, although the proliferation and mobilization of Lin-c-kit+Sca-1+ cells (LSK) was maintained following hematopoietic stress. These effects could be partially reversed by the selective PPARγ antagonist BADGE. Finally, we demonstrated in a co-culture system that differentiated adipocytes actively suppressed the myeloid differentiation of HSPCs.

**Conclusion:**

Taken together, our results demonstrate that rosiglitazone inhibits myeloid differentiation of HSPCs after stress partially by inducing bone marrow adipogenesis. Targeting the bone marrow microenvironment might be one mechanism by which rosiglitazone impairs stress-induced hematopoiesis.

## Introduction

Thiazolidinediones(TZDs) such as troglitazone, rosiglitazone and pioglitazone are peroxisome proliferator activated receptor-γ(PPARγ) agonists that improve glucose control in patients with type 2 diabetes by enhancing insulin sensitivity in target tissues. Though this group of drugs is usually well tolerated, they have been reported to cause several adverse effects, including hepatitis, edema, weight gain, bone loss and congestive heart failure [1].

Some hematological side effects, including anemia, leukopenia, thrombocytopenia, and pancytopenia, have been reported in patients receiving TZD treatment [2–4]. TZD-induced reductions in red blood cell count and hemoglobin(Hb) levels are traditionally considered to result from hemodilution effects[5].However, recent studies have demonstrated that the decreases in hematocrit and Hb are not correlated with changes in total body water or body weight [3,6]. One plausible explanation is that TZDs exert a suppressive effect on bone marrow, whereas other studies have shown that pretreatment with rosiglitazone for 5 days protects against 5-Fu-induced myelotoxity, which is FLT3-dependent [7–9]. Considering the clinical use of TZDs in diabetic patients, their long-term effects on homeostatic and stress hematopoiesis should be understood.

With regard to hematopoietic tissues, PPARγ is expressed in bone marrow stromal cells, CD34^+^ progenitor cells, normal monocyte/macrophages, megakaryocytes and neutrophils, indicating that PPARγ plays an essential role in both adipogenesis and hematopoiesis [10–12]. Despite having clearly defined roles in adipogenesis, the effect of TZDs on hematopoietic cells is unclear [13]. In some studies, PPARγ agonists have been recognized as inducers that increase the number of hematopoietic stem cells(HSCs) [14]. However, other studies have reported that 100–300μmol/l of TZDs slightly inhibits the growth of normal human hematopoietic cells [15,16]. PPARγ also negatively regulates the proliferation and differentiation of erythroid progenitor cells and pre-B cells [17,18].

Adequate hematopoiesis requires an intact and functional bone marrow microenvironment. Adipocytes are one of the most abundant cell types in the bone marrow niche and have received a great deal of attention because they are able to modulate hematopoiesis [19–21]. Recently, it was reported that adipocyte-rich bone marrow has fewer progenitor cells [19]. Using a mouse model with no fat in the bone marrow, researchers observed the improved engraftment of A-ZIP/F1 mouse bone marrow cells after primary and secondary transplantation, indicating that adipocytes might impair the engraftment of HSCs [19]. Long-term treatment with a PPARγ agonist activates adipocyte-specific gene expression and significantly enhances bone marrow adipogenesis [22–25]. Furthermore, our laboratory and others have demonstrated that BADGE, an inhibitor of PPARγ, can decrease marrow adiposity and improve hematopoietic recovery after chemotherapy or transplantation [19,20].Hence, we speculated that the PPARγ agonist rosiglitazone might inhibit hematopoietic recovery in response to stress by inducing bone marrow adipogenesis.

In this report, we treated mice with rosiglitazone for 6 weeks and examined the long-term effects of rosiglitazone on homeostatic and stress-induced hematopoiesis, and we found that rosiglitazone treatment delayed hematopoietic recovery and inhibited myelopoiesis after hematopoietic stress. We also found that rosiglitazone had no direct effect on the cellular phenotype or function of hematopoietic stem/progenitor cells(HSPCs). However, rosiglitazone-treated stromal cell lines showed enhanced potential to differentiate into adipocytes and inhibited the myeloid differentiation of co-cultured HSPC cells.

## Materials and Methods

### Reagents

Anti-mouse-Gr-1-APC, CD11b-APC, CD45R/B220-PE, and CD3-FITC were purchased from BioLegend (San Diego, CA, USA); anti-mouse-FcrR-FITC, CD34-PE, Sca-1-APC, c-kit-BV-421, IL-7R-PE-Cy7, biotin-conjugated lineage cocktail and APC-CY7-conjugated streptavidin were purchased from BD Bioscience (San Jose, CA, USA). A mouse hematopoietic progenitor cell enrichment kit was purchased from Stem Cell Technologies(Vancouver, Canada).

### Animals and treatment protocol

C57BL/6J female mice(6–8 weeks old) were provided by Beijing HFK Bioscience Co., Ltd. (Beijing, China). Mice were housed in a controlled environment with a 12 h light/dark cycle at 23°C (±2°C) and 40–50% relative humidity with free access to chow and standard water. The animal experiments were approved by the Animal Ethics Committee of Peking University Health Science Center (permit number: 2013–16). Measures to improve welfare assistance and endpoint criteria were established to minimize suffering and ensure animal welfare. Briefly, mice suffering severe infection or 30% weight loss were euthanized in accordance with our ethical guidelines. At each time point, mice were euthanized via cervical dislocation under sodium pentobarbital anesthesia (50 mg/kg).

The mice were randomly divided into three groups: (a) control(CTL) group,(b) rosiglitazone(ROSI) group, and (c) ROSI+BADGE group. To analyze the effect of rosiglitazone on hematopoiesis, the ROSI group mice were fed 5 g chow/d supplemented with 0.15 mg/g rosiglitazone maleate for 6 weeks[26].The control group mice were fed the same amount of non-supplemented chow. ROSI+BADGE group mice were given 60 mg/kg/d BADGE after 4 weeks on a rosiglitazone-enriched diet. To induce hematopoietic stress, all groups were injected once with 250 mg/kg 5-fluorouracil (5-Fu) intraperitoneally after 4 weeks on a normal diet or a rosiglitazone-enriched diet. The survival, food and rosiglitazone intake per cage as well as the body weight of individual animals were monitored daily.

### Histopathology

Mouse tibias were collected and fixed in 4% paraformaldehyde for 24 h. Tissues were decalcified in 20%(w/v) paraformaldehyde for 7 days at 4°C and were embedded in paraffin. Sections(4μm thick) were mounted on slides, deparaffinizedand stained with hematoxylin and eosin(HE). BM adipocytes were quantified as described previously [20,27].

### Peripheral blood cell and bone marrow mononuclear cell counts

Peripheral blood samples were collected in EDTA-coated tubes from the facial vein using lancets, and complete blood counts were analyzed using a Hemavet Model HV950hematology analyzer (Drew Scientific, UK). The bone marrow mononuclear cells(BMMNCs) were flushed from the long bones and counted using the hematology analyzer.

### Measurement of plasma glucose levels

Plasma glucose concentrations were determined at the beginning and at the end of the experiment using an Accu-Chek glucometer (Roche Diagnostics, Mannheim, Germany).

### Flow cytometric assays

Hematopoietic cells were collected from peripheral blood, spleen and bone marrow. The hematopoietic progenitor cells(HPCs: Lin-c-kit+Sca-1-), LSK cells(Lin-c-kit+Sca-1+), common lymphoid progenitors (CLP: Lin- c-kit low IL-7R+), common myeloid progenitors (CMP: Lin-FcγRlowCD34+), granulocyte/monocyte progenitors(GMP: Lin-FcγRhigh CD34+) and megakaryocyte/erythrocyte progenitors(MEP: Lin- FcγRlow CD34-) were analyzed as described previously [28].For cell cycle analysis, bone marrow cells were stained with the cell surface markers Sca-1-APC, Lin-cocktail-biotin, c-kit-BV421,and streptavidin-APC-cy7; washed; fixed in fixation/permeabilization working solution(eBioscience, San Diego, CA) for 45 min; washed again; and incubated in permeabilization buffer containing Ki-67-FITC for 30 min at room temperature. The cells were finally treated with 5 μl 7-AAD for 10 minutes before assessment.

### Colony-forming cell assay

CFC assays were performed in MethoCult GF M3434 methylcellulose with cytokines (Stem Cell Technologies, Canada) following the manufacturer’s instructions. The colonies were counted after 8–10 days using an inverted microscope(Olympus, Japan).

### Cell lines

The C3H10T1/2 cell line was obtained from the China Infrastructure Cell Line Resource, and the M2-10B4 cell line was obtained from the Shanghai Bioleaf Biotech Co.,Ltd. C3H10T1/2 was grown in MEM/EBSS supplemented with 10% fetal bovine serum(FBS). M2-10B4 was maintained in MEM supplemented with 10% FBS. To induce adipogenesis in the cell lines, confluent cultures were maintained in basic medium supplemented with 10μM rosiglitazone for 12 days. Adipogenic differentiation was confirmed by staining the lipid droplets with Oil Red O dye.

### PPARγantagonist treatment

PPARγ antagonists BADGE(20 μM)were added to C3H10T1/2 or M2-10B4 cells during adipogenic differentiation. Antagonists were pretreated for 24h before rosiglitazone was added. Dimethyl sulfoxide was used as a solvent for BADGE.

### Establishment of a co-culture system

Two stromal cell lines were plated in 24-well plates and cultured in basic medium in the presence or absence of 10 μM rosiglitazone. Lineage negative(Lin^-^) cells were selected using a mouse hematopoietic progenitor cell enrichment kit and were cultured on a layer of stromal cells in 500 μl of serum-free StemSpan SFEM medium (StemCell Technologies, Vancouver) supplemented with 100 ng/ml stem cell factor, 100 ng/ml Flt-3, and 20 ng/ml thrombopoietin. After 3 days of culture, the suspended cells were harvested and subjected to CFC assays and flow cytometric analysis.

### Cell proliferation assay

The cell proliferation rate was assayed by testing CCK-8. Briefly, 2×10^4^ Lin^-^ cells were seeded into 96-well culture plates and treated with PPARγ agonists for 7days. The OD value at 450 nm was evaluated using a microplate reader following the manufacturer’s instructions.

### RT-PCR

Total RNA was isolated from BMMNC using TRIzol Reagent(Invitrogen, USA). RNA (1μg)was reverse-transcribed using a High Capacity cDNA Reverse Transcription Kit (Applied Biosystems, USA) according to the manufacturer’s instructions. The cDNA samples were mixed with primers and SYBR Master Mix(Applied Biosystems, USA) to a total volume of 20μl. The data were analyzed using 7500 FAST System SDS version 2.0.6 software, and the changes in target gene expression were calculated using the comparative C_T_ method(fold change = 2^-ΔΔCt^) as described previously [29]. The primer sequences are shown in Table 1.

**Table 1:** Sequences of the primers used for qPCR.

Primers	Sequences(5’-3’)
PPARγ2 forward	CACTCGCATTCCTTTGACATC
PPARγ2 reverse	CGCACTTTGGTATTCTTGGAG
adiponectin forward	CGTCACTGTTCCCAATGTACC
adiponectin reverse	CGGAATGTTGCAGTAGAACTTG
PU.1 forward	CCCGGATGTGCTTCCCTTAT
PU.1 reverse	TCCAAGCCATCAGCTTCTCC
C/EBPα forward	CTCTCCACAAGGTTCATCAGG
C/EBPαreverse	GCTGTAGGTGCTTCCACTTCA
GAPDH forward	TCAATGACAACTTTGTCAAGCTCA
GAPDH reverse	GTGGGTGGTCCAGGGTTTCTTACT

## Statistics Analysis

All experiments were performed at least three times. The results were presented as the means± standard deviation(SD). The data were analyzed using Student’s *t*-test or ANOVA. A value of *P*<0.05 was considered statistically significant. All analyses were performed with GraphPad Prism (GraphPad Software, Inc. San Diego, CA).

## Results

### Rosiglitazone treatment has no effect on homeostatic hematopoiesis

**Fig 1:**
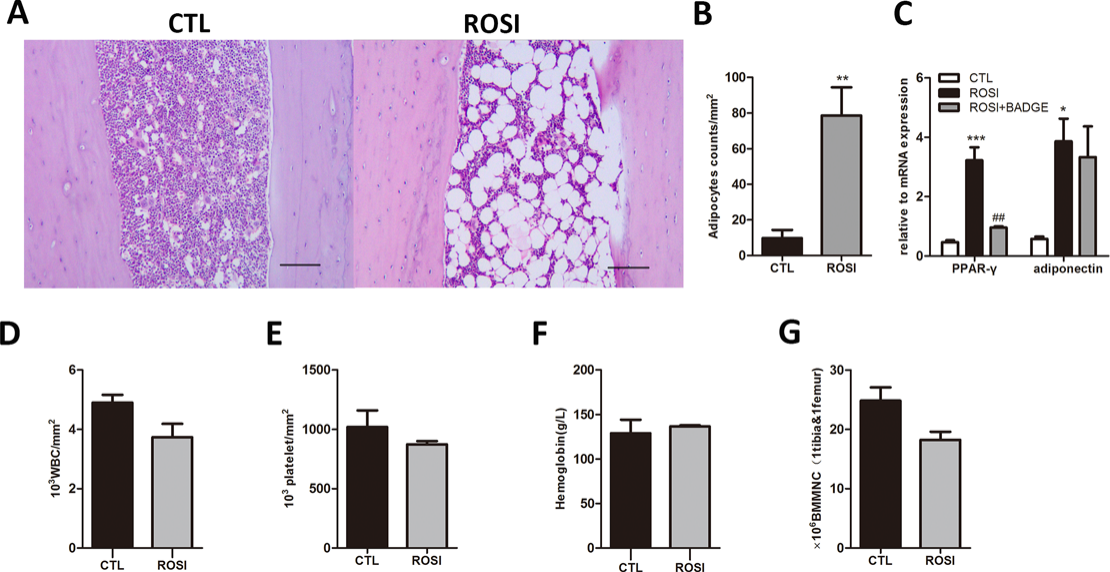
Normal homeostatic hematopoiesis in rosiglitazone-treated mice. (A) Adipocytes in tibia BM sections from rosiglitazone-treated and control mice (HE staining, scale bar 200 μm).(B) Adipocyte counts per mm^2^ in tibia BM sections from both groups of mice. (C) Expression of PPARγ2 and adiponectin in BMMNCs from the two groups of mice. (D-F) No significant differences in peripheral blood(PB) counts were observed in rosiglitazone-treated mice compared to the control mice. (G) A decreased bone marrow mononuclear cell(BMMNC) count was observed in rosiglitazone-treated mice compared to control mice, but this difference was not statistically significant(*P* = 0.066). The data are presented as the means ± SD from three independent experiments.

In our study, mice were fed a diet supplemented with rosiglitazone for six weeks to investigate the effect of rosiglitazone on homeostatic hematopoiesis. The cumulative dose of rosiglitazone calculated at the end of the experiment was 987.6 μg/g of body weight. In accordance with previous findings, we found that this treatment did not generate any differences regarding increases in weight (16.15±3.35%compared to 15.67±2.96%, n = 5, *P*>0.05) and glucose levels(188.28±23.42mg/dl compared to 192.24±27.52mg/dl, n = 5,*P*>0.05) [26].At the end of 6 weeks, increased numbers of adipocytes were observed in the long bone marrow of rosiglitazone-treated mice compared to the controls (Fig 1A and 1B). In addition, expression of the adipogenic marker genes PPARγ and adiponectin was also enhanced by rosiglitazone treatment(Fig 1C). However, rosiglitazone treatment did not cause any significant alteration of blood cellularity in normal, healthy mice (Fig 1D–1F; WBC 4900.00±458.26/mm^3^compared to 3733.33±776.75/mm^3^, n = 3, *P* = 0.089; PLT 1020000.00±242827.69/mm^3^compared to873333.33±47815.62/mm^3^, n = 3, *P* = 0.363;Hb129.00±26.21 g/l compared to136.67±2.08 g/l n = 3, *P* = 0.640). The average BM cellularity of mice in the rosiglitazone group was lower than that of the control group, but this difference was not statistically significant(18.21±2.40×10^6^ compared to 24.81±3.88×10^6^,n = 3, *P* = 0.066, Fig 1G).

### Rosiglitazone delays hematopoietic recovery after 5-Fu treatment

**Fig 2:**
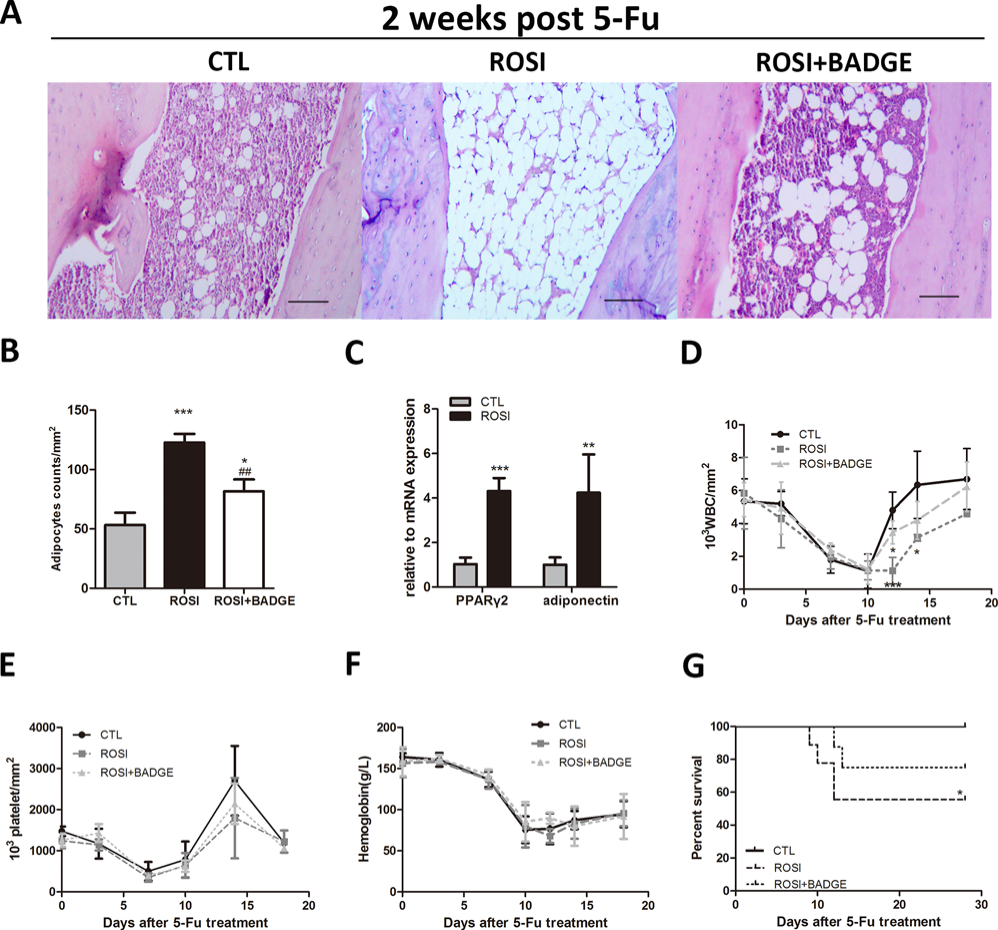
Delayed hematopoietic recovery and increased mortality after rosiglitazone treatment. (A)Adipocytes in tibia BM sections from the ROSI, the ROSI+BADGE and the control groups following 5-Fu treatment (HE staining, scale bar 200 μm). (B)Adipocyte counts per mm^2^ in tibia BM sections from the three groups of mice. (C)Expression of PPARγ2 and adiponectin in BMMNCs from the three groups of mice after 5-Fu treatment. (D) Six-week treatment with rosiglitazone significantly delayed the recovery of white blood cells after 5-Fu treatment, and this effect was reversed by administering the PPARγ antagonist BADGE. (E-F)No significant differences in platelet and Hb levels were observed in rosiglitazone-treated mice compared to control mice. (G) The survival of mice was assessed daily. The data represent the means±SD, n = 3 in A–C, n = 4 in D-F, n = 8 in G. *P<0.05 compared to CTL,***P<0.001 compared to CTL, ^##^*P*<0.01 compared to ROSI).

To investigate the effect of rosiglitazone on stress hematopoiesis, all mice were treated with 250mg/kg 5-Fu. After 2 weeks of treatment with 5-Fu, all mice exhibited adipocyte-rich bone marrow. Notably, mice in the rosiglitazone group had more adipocytes in their tibias, and the expression levels of PPARγ and adiponectin were increased in the rosiglitazone group compared to control mice, suggesting that rosiglitazone could also enhance adipogenesis following 5-Fu treatment(Fig 2A–2C).

In response to 5-Fu-induced hematopoietic stress, rosiglitazone-treated and control mice showed similar minimum values for WBCs, Hb and platelets. Upon recovery, however, the WBC counts in the rosiglitazone group increased more slowly. Platelet recovery was also slower in the rosiglitazone group, but this difference was not statistically significant (Fig 2D and 2E). Delayed hematopoietic recovery resulted in the death of 50% (n = 8, *P*<0.05) of the rosiglitazone-treated mice, whereas all control mice survived (Fig 2G). To exclude the possibility of death due to severe hypoglycemia, blood glucose levels were examined. We did not observe a significant difference in blood glucose levels between the ROSI group mice(182.16±22.82mg/dl) and the ROSI+BADGE group mice(187.8±42.68mg/dl)compared to the CTL group(186.30±15.45mg/dl). These data show that rosiglitazone treatment compromised the post-injury recovery of the hematopoietic system.

### Rosiglitazone treatment maintains LSK and inhibits myeloid progenitor cells after 5-Fu treatment

**Fig 3:**
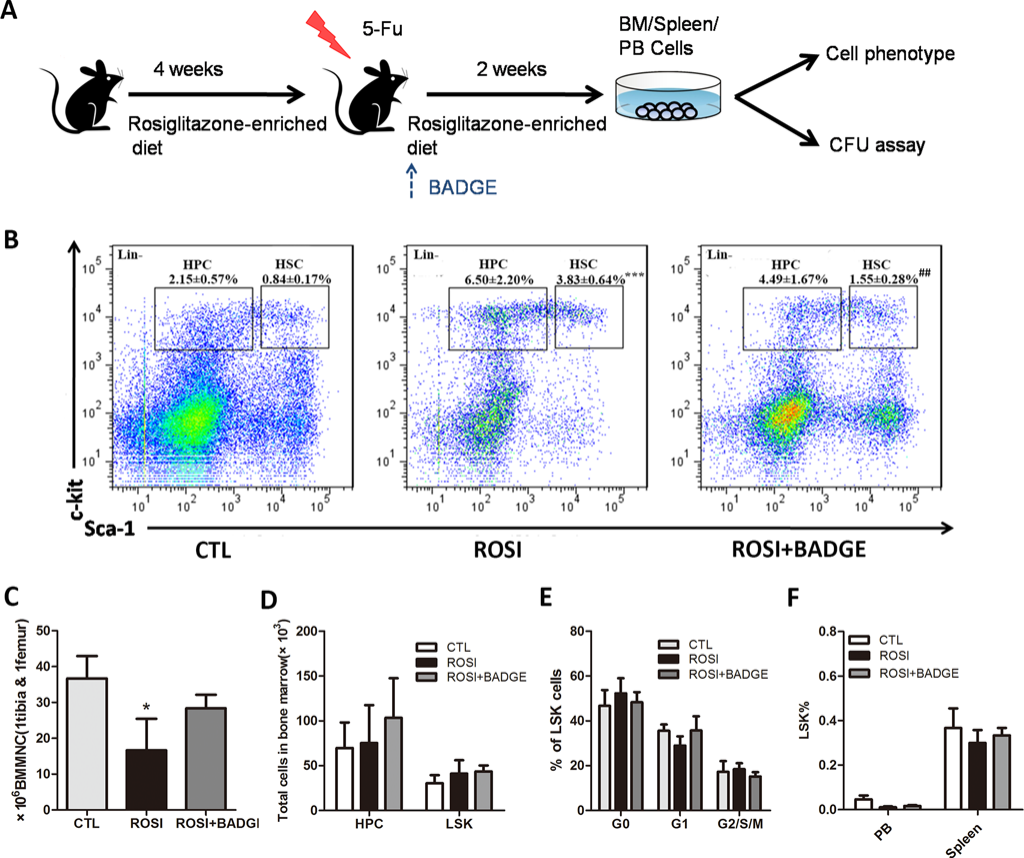
The *in vivo* effect of rosiglitazone treatment on LSK cells in bone marrow, peripheral blood and spleen under stress conditions. (A) Schematic of the experiment. In brief, the mice were fed a diet with or without rosiglitazone for 4 weeks. Then, they received a single dose of 250 mg/kg 5-Fu and continued their rosiglitazone-enriched or normal diet for two weeks. Two weeks after chemotherapy, the bone marrow, spleen and PB cells were isolated and used for flow cytometric analysis or a CFU assay. (B) Flow cytometric analyses of the LSK and HPC populations in the BM 14 days after 5-Fu. (C)The number of BMMNCs in rosiglitazone-treated mice was dramatically reduced compared to control mice 14 days after 5-Fu treatment. (D) The absolute number of HPC and LSK cells in the BM 14 days after 5-Fu treatment. (E)Cell cycle analysis of LSK in BM. (F)Flow cytometric analysis of the proportion of LSK cells in the PB and spleen 14 days after 5-Fu treatment. The data are presented as the means±SD of three independent experiments, *P<0.05 compared to CTL, ***P<0.001 compared to CTL, ^##^*P*<0.01 compared to ROSI.

To further explore the mechanism of delayed hematopoietic recovery, the phenotype of bone marrow hematopoietic cells after 5-Fu treatment was evaluated(Fig 3A). We observed that the rosiglitazone group exhibited an increased percentage of LSK compared to the control group mice on day14 after 5-Fu treatment(Fig 3B). Because the bone marrow cellularity of the rosiglitazone group mice was lower than that of the control mice, rosiglitazone treatment did not result in any significant alteration in the absolute number of LSK(Fig 3C and 3D). Furthermore, rosiglitazone treatment had no impact on the cell cycle of LSK cells (Fig 3E). Stress from cytotoxic agents caused by BM ablation promotes stem cell migration into a proliferative microenvironment such as the spleen and peripheral blood [30]. This is essential to reconstitute the stem cell pool and induce hematopoietic recovery. As shown in Fig 3F, there was no significant difference in LSK cell counts in PB and the spleen, suggesting that rosiglitazone treatment allows the maintenance of the LSK pool under stress conditions.

**Fig 4:**
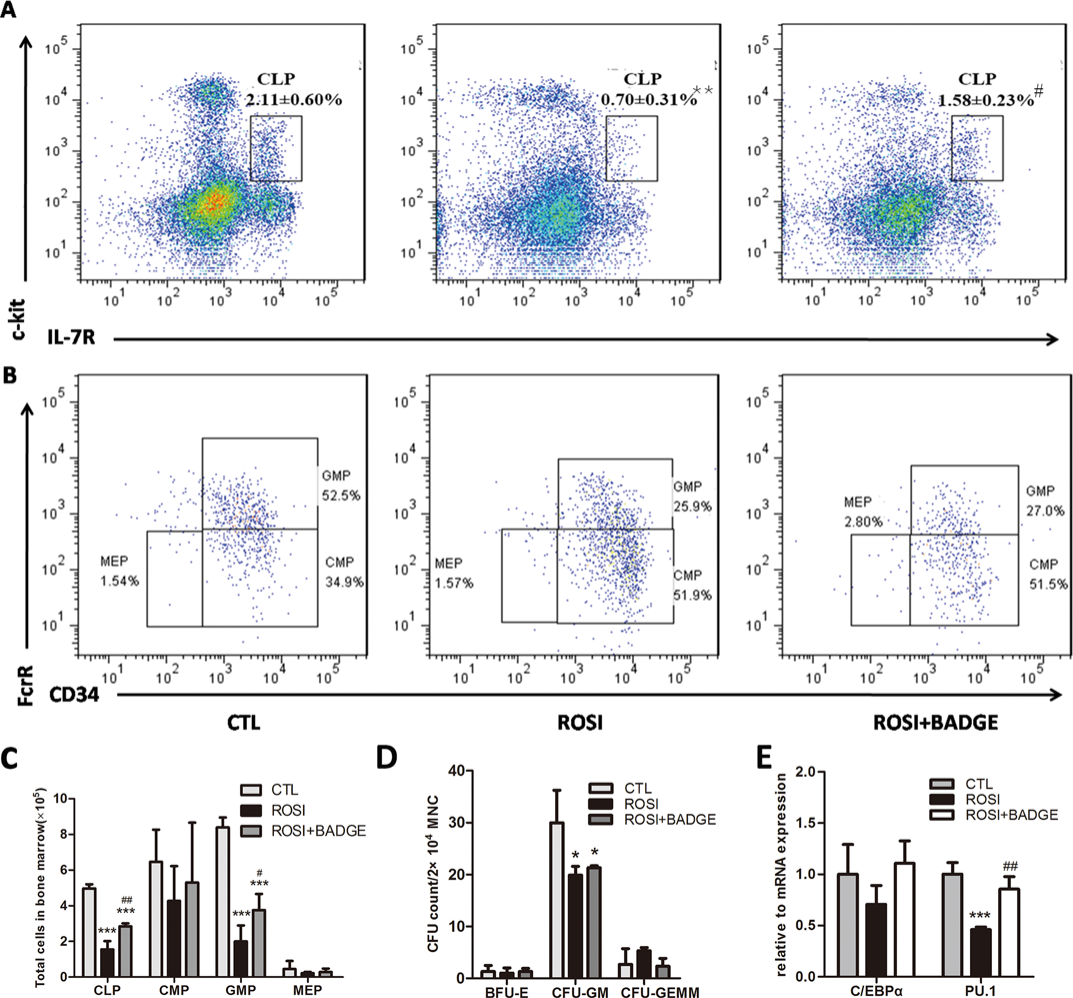
Rosiglitazone impairs differentiation of myeloid progenitors *in vivo*. (A-C) Flow cytometric analyses of HPC subsets in the BM 14 days after 5-Fu.(D) The colony-forming potential of BMMNCs 14 days after 5-Fu treatment. (E) RNA was extracted from BMMNCs from the CTL, ROSI and ROSI+BADGE group, and samples were analyzed for the expression of C/EBPα and PU.1. The data are presented as the means±SD of three independent experiments, *P<0.05 compared to CTL,**P<0.01 compared to CTL,^#^*P*<0.05compared to ROSI,^##^*P*<0.01 compared to ROSI.

Notably, the frequency and absolute number of GMP and CLP cells in the rosiglitazone group were dramatically decreased on day14 after 5-Fu treatment (Fig 4A–4C). Concomitantly, we observed a 1.5-fold reduction of CFU-GM in the rosiglitazone group, although the CFU-GEMM counts were not different (Fig 4D). Furthermore, qPCR analysis of PU.1 and C/EBPα, two transcription factors involved in myeloid differentiation, showed that PU.1 was expressed at significantly lower levels in BMMNCs from rosiglitazone-treated mice than in those from the controls(Fig 4E) [31]. The accumulation of cells in the primitive LSK and the loss of cells in the more differentiated GMP compartments indicate impaired myeloid development after rosiglitazone treatment.

### The effect of rosiglitazone on stress hematopoiesis is PPARγ-dependent

To demonstrate that the effect of rosiglitazone on stress hematopoiesis was PPARγ-dependent, we treated mice in the rosiglitazone group with a selective antagonist of PPARγ(BADGE) for two weeks. As shown in Fig 2A, bone marrow adipogenesis in the rosiglitazone group mice was markedly inhibited by BADGE treatment. The hematopoietic recovery and survival rate of the rosiglitazone group mice was improved by administering 60 mg/kg/d BADGE for two weeks (Fig 2D and 2G). Furthermore, the reduced CMP and GMP cell counts observed in rosiglitazone-treated mice was also partially reversed by BADGE(Fig 4A–4C). These data clearly suggest that rosiglitazone inhibits hematopoietic recovery after stress via a PPARγ-dependent mechanism. Furthermore, the data also indicate that impaired myeloid differentiation might be due to rosiglitazone-induced bone marrow adipogenesis.

### Rosiglitazone-treated stromal cells inhibit myeloid differentiation from HSPCs under stress condition

**Fig 5:**
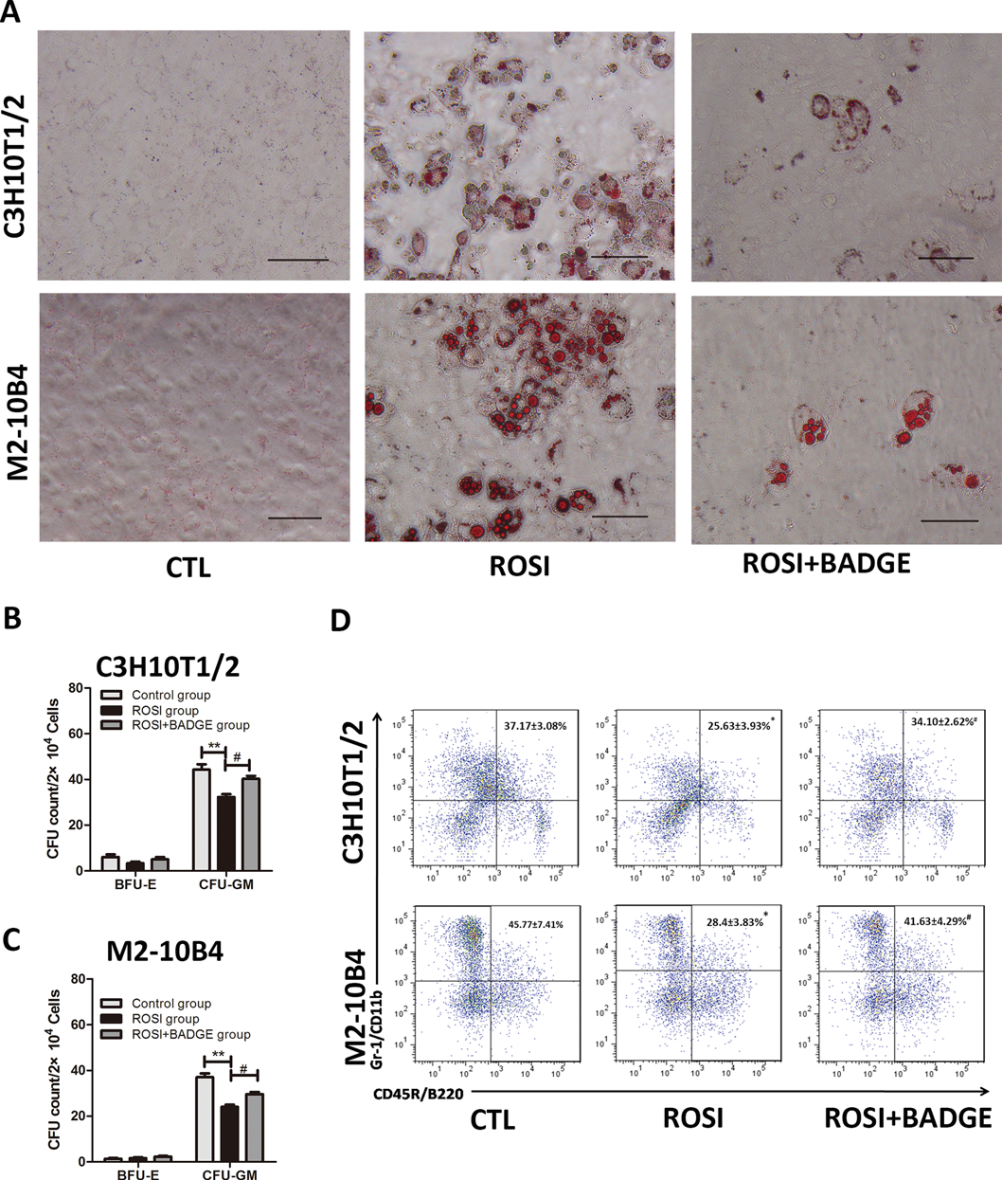
The effect of rosiglitazone-treated stromal cells on myeloid differentiation in HSPCs. (A) C3H10T1/2 cells and M2-10B4 were treated with 10 μM rosiglitazone in the presence or absence of BADGE(20μM). After 12 days of culture, the cells were fixed, and adipogenic differentiation was determined by Oil red O staining of the lipid droplets(scale bar 100 μm). (B, C) C3H10T1/2 cells and M2-10B4 were treated with 10 μM rosiglitazone in the presence or absence of BADGE(20μM) for 12 days. Then, the cells were washed twice with PBS followed by co-culture with Lin^-^ cells. After 3 days, colony-forming cell (CFC) assays (BFU-E and CFU-GM) were performed to determine the colony-forming viability of co-cultured Lin- cells. (D) The proportion of Gr-1^+^/CD11b^+^ cells was significantly decreased after co-culturing Lin^-^ cells with the rosiglitazone-treated C3H10T1/2 or M2-10B4 cell line. Furthermore, these effects were partially reversed by treating the stromal cells with BADGE. (The data are presented as the means±SD of three independent experiments, *P<0.05 vs. control, **P<0.01 vs. control, ^#^*P*<0.05vs. ROSI group).

Because rosiglitazone might impair hematopoiesis under stress, we used Lin- cells from 5-Fu-treated mice to test the direct and indirect effects of rosiglitazone on hematopoietic cells. First, we co-cultured Lin^-^ cells from d3 5-Fu bone marrow (bone marrow cells harvested 3 days after 5-FU administration)with rosiglitazone-treated stromal cells and detected the myeloid differentiation of HSPCs. As shown in Fig 5A, treatment with 10 μM rosiglitazone for 12 days clearly induced the adipogenic differentiation of M2-10B4 and C3H10T1/2 cells, an effect that was inhibited by BADGE treatment. To investigate the effect of rosiglitazone-treated stromal cells on myelopoiesis, flow cytometric and colony-forming assays were performed. Remarkably, the numbers of CFU-GM and Gr-1^+^/CD11b^+^ myeloid cells were significantly reduced after Lin^-^ cells were co-cultured with rosiglitazone-treated stromal cell lines for 3 days(Fig 5B–5D). Furthermore, these effects were partially reversed by treating the stromal cells with BADGE.

**Fig 6:**
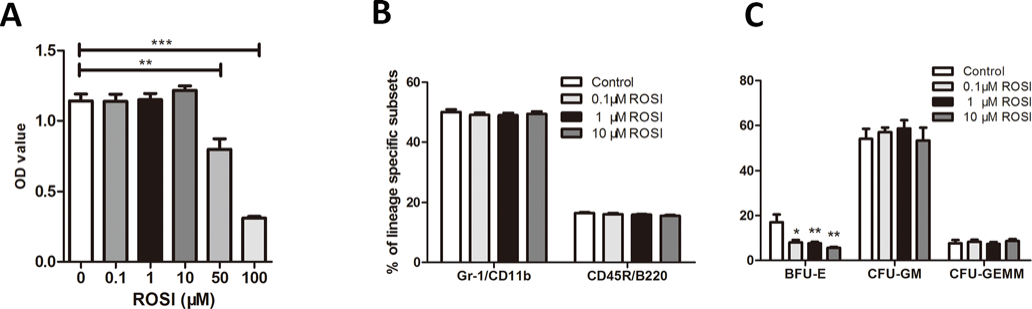
Rosiglitazone has no effect on hematopoietic progenitor cells *in vitro*. Lin^-^ cells from the BM of 5-Fu-treated mice were selected and cultured in the presence or absence of rosiglitazone for 7 days, and the cell proliferation, cell phenotype and colony-forming potential of Lin^-^ cells were assayed. The data are presented as the means±SD of three independent experiments, **P<0.01 compared to control, ***P<0.001compared to control.

To further determine whether rosiglitazone has a direct effect on HSPC myeloid differentiation under stress conditions, we treated Lin^-^ cells from d3 5-Fu bone marrow with different concentrations of rosiglitazone. The proliferation of Lin^-^ cells was unaffected at low concentrations of rosiglitazone (0.1 μM, 1 μM, and 10 μM) but was significantly inhibited at high concentrations(≥50 μM) (Fig 6A). Moreover, the survival rate was higher than 90% at low concentrations but significantly decreased at high concentrations, as assessed by trypan blue exclusion. Thus, subsequent experiments were performed using a low concentration of rosiglitazone. We observed that neither CFU-GM counts nor the proportion of Gr-1^+^/CD11b^+^ were affected by rosiglitazone, indicating that rosiglitazone might not directly inhibit the myeloid differentiation of HSPCs under stress conditions (Fig 6B and 6C). These results indicate that rosiglitazone impairs myelopoiesis partly by promoting adipogenesis rather than by inhibiting HSPCs directly.

## Discussion

TZDs have been used for many years as antidiabetic agents to improve hyperglycemia and hyperlipidemia. These drugs bind and activate the nuclear receptor PPARγ, which exerts critical control over the adipogenic differentiation process. Some studies have suggested that in addition to inducing adipogenic differentiation, TZDs also exert suppressive effects on hematopoiesis [2,6]. However, the potential effect of long-term TZD treatment on hematopoiesis *in vivo* is poorly documented. Our study revealed that the PPARγ agonist rosiglitazone can inhibit myeloid differentiation without compromising HSPC function, which might ultimately lead to delayed hematopoietic recovery after stress. Moreover, the effect of TZDs on myeloid differentiation is partly due to an indirect effect on the bone marrow microenvironment rather than a direct effect on HSPCs.

Although TZDs have some adverse hematopoietic effects in diabetic patients, we did not find that rosiglitazone inhibited homeostatic hematopoiesis in normal mice, which was consistent with previously published results [32]. HSPCs are more easily impaired by certain factors under stress conditions. Thus, it was reasonable to observe that hematopoietic recovery in the rosiglitazone group was delayed after 5-Fu treatment. We further assessed the subsets of HSPCs and found that the absolute number and cell cycle of LSK cells were not affected. Because LSK cells are approximately 10% HSC in addition to progenitors, our study suggests that rosiglitazone does not affect HSPC function[33]. However, it is possible that the function of LSKs might be impaired by rosiglitazone after serial 5-Fu injection as HSPCs that have started to cycle become more vulnerable to the drug after a second dose 5-Fu[34].We found that the frequency and absolute number of GMPs and CLPs were decreased. Moreover, CFU-GM counts and the expression of PU.1 in the bone marrow were also significantly decreased in the rosiglitazone group compared to the control group, which further confirmed defective myelopoiesis induced by rosiglitazone. It should be noted that there was no difference in the expression of C/EBPα between the rosiglitazone group mice and the controls. C/EBPα is part of a family of leucine zipper transcription factors and plays an important role in myeloid differentiation and adipogenesis. Mutation of the C/EBPα gene results in a selective block to the differentiation of neutrophils, and reduced expression of C/EBPα might cause defective neutrophilic differentiation [31,35]. The C/EBPα gene can also be regulated by PPARγ agonists[36]; thus, the C/EBPα level in rosiglitazone-treated mice might not accurately reflect the process of myelopoiesis. These data indicate that rosiglitazone might impair the myeloid differentiation of HSPCs in response to stress, which is a plausible mechanism for the increase in undifferentiated HSPCs observed in our experiment.

In contrast to our results, other studies have shown that pretreatment with rosiglitazone for 5 days leads to an accelerated recovery of hematopoiesis following 5-Fu administration [8,9]. These discrepancies can be explained in several ways. The most important of all possible explanations is that the rosiglitazone dosage and treatment duration differed between different studies. The conditions used in this study significantly increased bone marrow adipocyte counts, and bone marrow adipocytes negatively regulate hematopoiesis. Differences in insulin levels could be another possible explanation. Insulin is a growth factor for hematopoiesis, and serum insulin tends to increase after 5days of rosiglitazone treatment, whereas it decreases after long-term treatment with rosiglitazone [26]. Other factors, such as the severity of the stress, might also explain the observed differences.

PPARγ agonists play a pivotal role in regulating the proliferation and differentiation of leukemic cell lines and hematopoietic cells [15,37]. These drugs suppress cellular growth by inducing apoptosis in leukemia cell lines, and they have a mildly toxic effect on normal hematopoietic cells. Furthermore, PPARγ agonists inhibit the maturation and proliferation of primary erythroid progenitor cells and rapidly induce apoptosis in B cells [15,17]. However, other studies have shown that TZD treatment can increase the number of hematopoietic stem cells in bone marrow and peripheral blood when combined with a hematopoietic stem cell mobilizer [14]. We observed that the optimized concentration of rosiglitazone did not affect the cell phenotype and CFU-GM counts of HSPCs under stress. Thus, the effect of rosiglitazone on myelopoiesis cannot be explained by a direct effect, and other mechanisms must be involved.

PPARγ agonists are strong stimulators of adipogenesis. In line with previous reports, we found that long-term rosiglitazone treatment increased bone marrow fat in mice, which was accompanied by a delay in hematopoietic recovery[26]. As adipocytes are known to be a negative regulator of bone marrow cells, the increase in fat space after rosiglitazone treatment prompted us to ask whether adipocytes inhibit the myeloid differentiation of HSPCs, thereby contributing to delayed hematopoietic recovery. We found that the effect of rosiglitazone on stress hematopoiesis could be partially reversed by the PPARγ inhibitor BADGE, which has been shown to prevent bone marrow adipocyte formation *in vivo* and *in vitro*[19,20,38]. BADGE treatment also increased the absolute number of GMPs in rosiglitazone-treated mice, while it did not rescue the effects of rosiglitazone on CFU-GM counts. However, if CFU numbers were converted to total numbers in the BM, the total number of CFU-GM counts in BADGE group mice was significantly higher than ROSI group mice. Furthermore, rosiglitazone promoted the differentiation of two stromal cell lines into adipocytes, and rosiglitazone-treated stromal cell lines significantly inhibited the myeloid differentiation of co-cultured HSPCs *in vitro*, an effect that was also reversed by BADGE treatment. Thus, it is reasonable that rosiglitazone inhibits myelopoiesis in part by promoting bone marrow adipogenesis.

Other mechanisms might also be involved in the effects of rosiglitazone on stress hematopoiesis. PPARγ agonists can inhibit osteoblast differentiation and osteoclast function[25, 39]. It has been reported that the osteoblastic lineage plays a central role establishing the HSC niche. Osteoblastic cells are crucial players for the homeostasis of hematopoiesis as they express several cell-to-cell receptors (e.g., N-Cadherin, Jagged, VCAM-1), soluble and cell-surface associated cytokines and growth factors that are essential for normal HSC function [40,41]. It is possible that rosiglitazone treatment negatively impacts myelopoiesis by suppressing osteoblast differentiation. However, the role of osteoclasts in the maintenance of hematopoietic stem cells is still controversial. Osteoclasts are reportedly required for hematopoietic stem and progenitor cell mobilization. In contrast, other studies have suggested that osteoclasts are dispensable for HSC mobilization and might function as negative regulators in the hematopoietic system[42].

In conclusion, our results show that rosiglitazone inhibits myelopoiesis through its action on the bone marrow microenvironment, which can delay hematopoietic recovery. These results suggest a plausible mechanism for the impaired hematopoiesis observed in patients receiving TZD treatment and provide new evidence for the benefits of PPARγ inhibitors that are used to improve hematopoietic recovery. Furthermore, the model of adipocyte hyperplasia induced by rosiglitazone might help us understand the mechanism of adipocyte-imposed hematopoietic inhibition.

## References

[1] Ahmadian M, Suh JM, Hah N, Liddle C, Atkins AR, Downes M, et al PPARγ signaling and metabolism: the good, the bad and the future. Nat Med. 2013;19: 557–566. 10.1038/nm.3159 23652116 PMC3870016

[2] Clevidence DE, Juckett MB, Lucarelli MJ. Marrow suppression with myelodysplastic features, hypoerythropoetinemia, and lipotrophicproptosis due to rosiglitazone. WMJ. 2009;108: 462–465. 20131689

[3] Berria R, Gastaldelli A, Lucidi S, Belfort R, Defilippis E, Easton C, et al Reduction in hematocrit level after pioglitazone treatment is correlated with decreased plasma free testosterone level, not hemodilution, in women with polycystic ovary syndrome. Clin Pharmacol Ther. 2006;80: 105–114. 10.1016/j.clpt.2006.03.014 16890572

[4] Karakurt F, Kargili A, Kasapoglu B. Pioglitazone induced reversible pancytopenia. Exp Clin Endocrinol Diabetes. 2010;118: 96–97. 10.1055/s-0029-1234065 19834871

[5] Tang WHW, Francis GS, Hoogwerf BJ, Young JB. Fluid retention after initiation of thiazolidinedione therapy in diabetic patients with established chronic heart failure. J Am Coll Cardiol. 2003;41: 1394–1398. 10.1016/S0735-1097(03)00159-1 12706937

[6] Lin KD, Lee MY, Feng CC, Chen BK, Yu ML, Shin SJ. Residual effect of reductions in red blood cell count and haematocrit and haemoglobin levels after 10-month withdrawal of pioglitazone in patients with Type 2 diabetes. Diabet Med. 2014;31: 1341–1349. 10.1111/dme.12481 24797920

[7] Djazayeri K, Szilvássy Z, Peitl B, Németh J, Nagy L, Kiss A, et al Accelerated recovery of 5-fluorouracil-damaged bone marrow after rosiglitazone treatment. Eur J Pharmacol. 2005;522: 122–129. 10.1016/j.ejphar.2005.08.053 16213483

[8] Géresi K, Benkő K, Szabó B, Megyeri A, Peitl B, Szilvássy Z, et al Toxicity of cytotoxic agents to granulocyte–macrophage progenitors is increased in obese Zucker and non-obese but insulin resistant Goto-Kakizaki rats. Eur J Pharmacol. 2012;696: 172–178. 10.1016/j.ejphar.2012.09.018 23022328

[9] Avagyan S, Aguilo F, Kamezaki K, Snoeck H-W. (2011). Quantitative trait mapping reveals a regulatory axis involving peroxisome proliferator-activated receptors, PRDM16, transforming growth factor- 2 and FLT3 in hematopoiesis. Blood. 2011;118: 6078–6086. 10.1182/blood-2011-07-365080 21967974 PMC3234666

[10] Konopleva M, Andreeff M. Role of peroxisome proliferator-activated receptor-γ in hematologic malignancies. Curr Opin Hematol. 2002;9: 294–302. 10.1097/00062752-200207000-00006 12042703

[11] Zang C, Liu H, Posch MG, Waechter M, Facklam M, Fenner MH, et al Peroxisome proliferator-activated receptor γ ligands induce growth inhibition and apoptosis of human B lymphocytic leukemia. Leuk Res. 2004;28: 387–397. 10.1016/j.leukres.2003.07.005 15109539

[12] Akbiyik F, Ray DM, Gettings KF, Blumberg N, Francis CW, Phipps RP. Human bone marrow megakaryocytes and platelets express PPAR, and PPAR agonists blunt platelet release of CD40 ligand and thromboxanes. Blood. 2004;104: 1361–1368. 10.1182/blood-2004-03-0926 15130939

[13] Campeau PM, Astapova O, Martins R, Bergeron J, Couture P, Hegele RA, et al Clinical and molecular characterization of a severe form of partial lipodystrophy expanding the phenotype of PPAR deficiency. J Lipid Res. 2012;53: 1968–1978. 10.1194/jlr.P025437 22750678 PMC3413236

[14] Nagasawa T, Sugiyama T, Omatsu Y. Inducer composition for hematopoietic stem cells. Patent WO2011030847-A1. 10 Sep 2010.

[15] Saiki M, Hatta Y, Yamazaki T, Itoh T, Enomoto Y, Takeuchi J, et al Pioglitazone inhibits the growth of human leukemia cell lines and primary leukemia cells while sparing normal hematopoietic stem cells. Int J Oncol. 2006;29: 437–443. 10.3892/ijo.29.2.437 16820887

[16] Prost S, Le Dantec M, Augé S, Le Grand R, Derdouch S, Auregan G, et al Human and simian immunodeficiency viruses deregulate early hematopoiesis through a Nef/PPAR gamma/STAT5 signaling pathway in macaques. J Clin Invest. 2008;118: 1765–1775. 10.1172/JCI33037 18431514 PMC2323187

[17] Nagasawa E, Abe Y, Nishimura J, Yanase T, Nawata H, Muta K. Pivotal role of peroxisome proliferator–activated receptor γ (PPARγ) in regulation of erythroid progenitor cell proliferation and differentiation. Exp Hematol. 2005;33: 857–864. 10.1016/j.exphem.2005.05.003 16038777

[18] Schlezinger JJ, Jensen BA, Mann KK, Ryu H-Y, Sherr DH. Peroxisome proliferator-activated receptor γ-mediated NF-κB activation and apoptosis in pre-B cells. J Immunol. 2002;169: 6831–6841. 10.4049/jimmunol.169.12.6831 12471115

[19] Naveiras O, Nardi V, Wenzel PL, Hauschka PV, Fahey F, Daley GQ. Bone-marrow adipocytes as negative regulators of the haematopoietic microenvironment. Nature. 2009;460: 259–263. 10.1038/nature08099 19516257 PMC2831539

[20] Zhu R, Wu M, Li Z, Zhang Y, Liu K. Hematopoietic recovery following chemotherapy is improved by BADGE-induced inhibition of adipogenesis. Int J Hematol. 2013;97: 58–72. 10.1007/s12185-012-1233-4 23264188

[21] Belaid-Choucair Z, Lepelletier Y, Poncin G, Thiry A, Humblet C, Maachi M, et al Human bone marrow adipocytes block granulopoiesis through neuropilin-1-induced granulocyte colony-stimulating factor inhibition. Stem Cells. 2008;26: 1556–1564. 10.1634/stemcells.2008-0068 18388301

[22] Sottile V, Seuwen K, Kneissel M. Enhanced marrow adipogenesis and bone resorption in estrogen-deprived rats treated with the PPARgamma agonist BRL49653 (rosiglitazone). Calcif Tissue Int. 2004;75: 329–337. 10.1007/s00223-004-0224-8 15549648

[23] Sadie-Van Gijsen H, Hough FS, Ferris WF. Determinants of bone marrow adiposity: the modulation of peroxisome proliferator-activated receptor-γ2 activity as a central mechanism. Bone. 2013;56: 255–265. 10.1016/j.bone.2013.06.016 23800517

[24] Beck GR, Khazai NB, Bouloux GF, Camalier CE, Lin Y, Garneys LM, et al The effects of thiazolidinediones on human bone marrow stromal cell differentiation in vitro and in thiazolidinedione-treated patients with type 2 diabetes. Transl Res J Lab Clin Med. marzo de 2013;161(3):145–55. 10.1016/j.trsl.2012.08.006 PMC3546231 23022285

[25] Patel JJ, Butters OR, Arnett TR. PPAR agonists stimulate adipogenesis at the expense of osteoblast differentiation while inhibiting osteoclast formation and activity. Cell Biochem Funct. junio de 2014;32(4):368–77. 10.1002/cbf.3025 24615887

[26] Lazarenko OP, Rzonca SO, Hogue WR, Swain FL, Suva LJ, Lecka-Czernik B. Rosiglitazone induces decreases in bone mass and strength that are reminiscent of aged bone. Endocrinology. 2007;148: 2669–2680. 17332064 10.1210/en.2006-1587 PMC2084459

[27] Wang W, Zhang Y, Lu W, Liu K. Mitochondrial reactive oxygen species regulate adipocyte differentiation of mesenchymal stem cells inhematopoietic stress induced by arabinosylcytosine. PLOS ONE. 2015;10: e0120629 10.1371/journal.pone.0120629 25768922 PMC4359087

[28] Frascoli M, Proietti M, Grassi F. Phenotypic analysis and isolation of murine hematopoietic stem cells and lineage-committed progenitors. J Vis Exp. 2012;65: 3736 10.3791/3736 22805770 PMC3471276

[29] Schmittgen TD, Livak KJ. Analyzing real-time PCR data by the comparative C(T) method. Nat Protoc. 2008;3: 1101–1108. 10.1038/nprot.2008.73 18546601

[30] Zhao M, Ross JT, Itkin T, Perry JM, Venkatraman A, Haug JS, et al FGF signaling facilitates postinjury recovery of mouse hematopoietic system. Blood. 2012;120: 1831–1842. 10.1182/blood-2011-11-393991 22802336 PMC3433089

[31] Rodriguez S, Chora A, Goumnerov B, Mumaw C, Goebel WS, Fernandez L, et al Dysfunctional expansion of hematopoietic stem cells and block of myeloid differentiation in lethal sepsis.Blood. 2009;114: 4064–4076. 10.1182/blood-2009-04-214916 19696201 PMC2774548

[32] Spindler TJ, Tseng AW, Zhou X, Adams GB. Adipocytic cells augment the support of primitive hematopoietic cells in vitro but have no effect in the bone marrow niche under homeostatic conditions. Stem Cells Dev. 2014;23: 434–441. 10.1089/scd.2013.0227 24083324 PMC3996940

[33] Challen GA, Boles N, Lin KK-Y, Goodell MA. Mouse hematopoietic stem cell identification and analysis. Cytometry A. 2009;75:14–24. 10.1002/cyto.a.20674 19023891 PMC2640229

[34] Harrison DE, Lerner CP. Most primitive hematopoietic stem cells are stimulated to cycle rapidly after treatment with 5-fluorouracil. Blood. 1991;78:1237–1240. 1878591

[35] Zhang DE, Zhang P, Wang ND, Hetherington CJ, Darlington GJ, Tenen DG. Absence of granulocyte colony-stimulating factor signaling and neutrophil development in CCAAT enhancer binding protein alpha-deficient mice. Proc Natl Acad Sci U S A. 1997;94: 569–574. 10.1073/pnas.94.2.569 9012825 PMC19554

[36] Wu Z, Rosen ED, Brun R, Hauser S, Adelmant G, Troy AE, et al Cross-regulation of C/EBP alpha and PPAR gamma controls the transcriptional pathway of adipogenesis and insulin sensitivity. Mol Cell. 1999;3: 151–158. 10.1016/S1097-2765(00)80306-8 10078198

[37] Abbasi P, Shamsasenjan K, Movassaghpour Akbari AA, Akbarzadehlaleh P, Dehdilani N, Ejtehadifar M. The effect of Baicalin as A PPAR activator on erythroid differentiation of CD133(+)hematopoietic stem cells in umbilical cord blood. Cell J. 2015;17:15–26. 25870831 10.22074/cellj.2015.508 PMC4393663

[38] Botolin S, McCabe LR. Inhibition of PPARgamma prevents type I diabetic bone marrow adipositybut not bone loss. J Cell Physiol. 2006; 209:967–976. 16972249 10.1002/jcp.20804

[39] Sadie-Van Gijsen H, Hough FS, Ferris WF. Determinants of bone marrow adiposity: the modulation of peroxisome proliferator-activated receptor-γ2 activity as a central mechanism. Bone. octubre de 2013;56:255–265. 10.1016/j.bone.2013.06.016 23800517

[40] Arai F, Hirao A, Ohmura M, Sato H, Matsuoka S, Takubo K, et al Tie2/Angiopoietin-1 Signaling Regulates Hematopoietic Stem Cell Quiescence in the Bone MarrowNiche. Cell. 2004; 118:149–161. 15260986 10.1016/j.cell.2004.07.004

[41] Calvi LM, Adams GB, Weibrecht KW, Weber JM, Olson DP, Knight MC, et al Osteoblastic cells regulate thehaematopoietic stem cell niche. Nature. 2003; 425:841–846. 14574413 10.1038/nature02040

[42] Miyamoto T.Role of osteoclasts in regulating hematopoietic stem and progenitor cells. World J Orthop. 2013 10 18;4(4):198–206. 10.5312/wjo.v4.i4.198 24147255 PMC3801239

